# Cardiovascular outcomes and safety of SGLT2 inhibitors in chronic kidney disease patients

**DOI:** 10.3389/fendo.2023.1236404

**Published:** 2023-11-16

**Authors:** Xiutian Chen, Jiali Wang, Yongda Lin, Kaijin Yao, Yina Xie, Tianbiao Zhou

**Affiliations:** Department of Nephrology, The Second Affiliated Hospital of Shantou University Medical College, Shantou, China

**Keywords:** sodium-glucose cotransporter 2(SGLT2) inhibitors, chronic kidney disease (CKD), randomized controlled trial (RCT), meta-analysis, cardiovascular

## Abstract

**Background:**

Sodium–glucose co-transporter 2 (SGLT2) inhibitors provide cardiovascular protection for patients with heart failure (HF) and type 2 diabetes mellitus (T2DM). However, there is little evidence of their application in patients with chronic kidney disease (CKD). Furthermore, there are inconsistent results from studies on their uses. Therefore, to explore the cardiovascular protective effect of SGLT2 inhibitors in the CKD patient population, we conducted a systematic review and meta-analysis to evaluate the cardiovascular effectiveness and safety of SGLT2 inhibitors in this patient population.

**Method:**

We searched the PubMed® (National Library of Medicine, Bethesda, MD, USA) and Web of Science™ (Clarivate™, Philadelphia, PA, USA) databases for randomized controlled trials (RCTs) of SGLT2 inhibitors in CKD patients and built the database starting in January 2023. In accordance with our inclusion and exclusion criteria, the literature was screened, the quality of the literature was evaluated, and the data were extracted. RevMan 5.3 (The Nordic Cochrane Centre, The Cochrane Collaboration, Copenhagen, Denmark) and Stata® 17.0 (StataCorp LP, College Station, TX, USA) were used for the statistical analyses. Hazard ratios (HRs), odds ratios (ORs), and corresponding 95% confidence intervals (CIs) were used for the analysis of the outcome indicators.

**Results:**

Thirteen RCTs were included. In CKD patients, SGLT2 inhibitors reduced the risk of cardiovascular death (CVD) or hospitalization for heart failure (HHF) by 28%, CVD by 16%. and HHF by 35%. They also reduced the risk of all-cause death by 14% without increasing the risk of serious adverse effects (SAEs) and urinary tract infections (UTIs). However, they increased the risk of reproductive tract infections (RTIs).

**Conclusion:**

SGLT2 inhibitors have a cardiovascular protective effect on patients with CKD, which in turn can significantly reduce the risk of CVD, HHF, and all-cause death without increasing the risk of SAEs and UTIs but increasing the risk of RTIs.

## Introduction

Diabetes is a chronic metabolic disease, and type 2 diabetes mellitus (T2DM) is the most common. According to statistics, in 2021, the prevalence of diabetes among people aged 20 to 79 worldwide was estimated to be 10.5% (536.6 million people). This is expected to rise to 12.2% (783.2 million people) by 2045. The prevalence of diabetes is similar between men and women, with the highest prevalence occurring in those between the ages of 75 and 79. The 2021 global diabetes-related health expenditure was about 966 billion US dollars. It is estimated that the expenditure will reach 105.4 billion US dollars by 2045. At present, more than 500 million people in the world suffer from diabetes, which means that more than 10.5% of the world’s adults suffer from this disease (1). The number of diabetic patients will continue to increase rapidly in the future.

Chronic kidney disease (CKD) is defined as having a low glomerular filtration rate or high proteinuria, which affects 15%–20% of adults worldwide. Diabetes is the main cause of CKD, while hyperglycemia and CKD are the main risk factors for cardiovascular disease (CVD) and total mortality. CKD increases the risk of various adverse consequences, but CVD is particularly important because it is the main cause of death in this clinical population. CKD is associated with a variety of CVD results, including coronary heart disease, stroke, peripheral arterial disease, arrhythmia, heart failure, and venous thrombosis. It is worth noting that CKD is particularly related to serious CVD outcomes, such as cardiovascular mortality and heart failure (2).

Sodium–glucose co-transporter 2 (SGLT2) is the dominant transporter in sodium–glucose co-transporters that mediate the process of renal reabsorption of glucose. SGLT2, mainly distributed in the S1 segment of the renal proximal tubule, is a transporter with low affinity and high transport capacity, and its main physiological function is to complete the reabsorption of 90% of the glucose in the glomerular filtration fluid in the renal proximal tubule (3, 4). SGLT2 inhibitors are a class of antihyperglycemic drugs approved for the treatment of T2DM. These drugs block the reabsorption of glucose in the kidneys by inhibiting SGLT2, thus increasing urinary glucose excretion, promoting urination, lowering blood sugar levels, and improving blood sugar control in an insulin-independent way. SGLT2 inhibitors can block glucose reabsorption in proximal renal tubules, and they have many beneficial effects, including reducing body weight and serum uric acid, lowering blood pressure levels, and weakening glomerular hyperfiltration, which may be related to urinary sodium excretion with diabetes. In addition to lowering blood pressure, SGLT2 inhibitors have other protective properties that may be related to the cardiovascular outcomes of diabetic nephropathy.

Due to the high level of expression of SGLT2, sodium–glucose reabsorption in proximal renal tubules is increased, which leads to a decrease in sodium ions reaching the dense spots of the distal renal tubules, in turn leading to the dilation of afferent arterioles. Therefore, the glomerulus presents high perfusion, high internal pressure, and high filtration. SGLT2 inhibitors can reduce renal hyperfiltration, activate glomerular feedback contraction into glomerular arterioles, and reduce intraglomerular pressure through the mechanism of increasing sodium secretion. SGLT2 inhibition has also been proven to prevent renal hyperfiltration by lowering blood pressure levels and glomerular size and inhibiting renal growth factors (5). SGLT2 inhibitors can reduce the increase in systolic blood pressure in the treatment of T2DM. Moreover, the effects of dapagliflozin and canagliflozin can stabilize the changes in patients’ levels of triglycerides. This shows that SGLT2 inhibitors can not only lower blood sugar levels but also improve blood pressure and blood lipid levels through the lowering of blood sugar levels (6). It has been shown that SGLT2 inhibitors combined with an angiotensin-converting enzyme inhibitor upregulate the renin–angiotensin system effect in nephropathy, therefore suggesting that blood pressure changes may be influenced by SGLT2 inhibitors (7, 8).

Many studies have shown that the use of SGLT2 inhibitors can reduce the risk of severe cardiac and renal prognosis, such as reducing the risk of vascular death and hospitalization for heart failure (HHF) (9–11). However, experimental subgroups have analyzed the cardiovascular protective effect of SGLT2 inhibitors on CKD patients and found that the results are inconsistent (12–15).

In summary, the prevalence of CKD is high, and diabetes is the most common cause, which often occurs together with hypertension. SGLT2 inhibitors have a cardiovascular protective effect in patients with diabetes and heart failure. However, clinical trial results are inconsistent regarding the level of cardiovascular protection of SGLT2 inhibitors for patients with CKD. Therefore, this study will comprehensively discuss the cardiovascular benefits and safety of SGLT2 inhibitors for patients with CKD, so as to provide evidence for the clinical application of SGLT2 inhibitors.

## Materials and methods

### Search strategy

We searched the PubMed® (National Library of Medicine, Bethesda, MD, USA) and Web of Science™ (Clarivate™, Philadelphia, PA, USA) databases; the search range was from the establishment of each database to January 2023. The keywords were diabetes neuropathies, randomized controlled trial, and sodium-glucose co-transporter-2 inhibitor. The recall rate was improved by searching American clinical research centers and reading the related references included in the literature.

### Inclusion and exclusion criteria

Inclusion criteria were as follows: (1) a randomized controlled trial (RCT), which was randomized, double-blind, and controlled; (2) some of the patients met the diagnostic criteria for CKD of an estimated glomerular filtration rate (eGFR) of < 60 mL/min/1.73m^2^; (3) the experimental group was given SGLT2 inhibitors and the control group was given placebo; (4) effectiveness indicators of the incidence of cardiovascular death (CVD), heart failure hospitalisation (HFF), or all-cause deaths; and (5) safety indicators of the incidence of serious adverse effects (SAEs), reproductive tract infections (RTIs), and urinary tract infections (UTIs).

Exclusion criteria were as follows: (1) no complete test design and defects in the test design; (2) being a Phase II clinical study; (3) the repeated publication of research; and (4) no such validity index at the end of the experiment.

### Outcome measures

After selecting the effect amount, two researchers independently extracted the input data and then exchanged and compared the data after completion. After data reconciliation, a meta-analysis was performed using Stata 17.0 (STATA.MP; StataCorp LP, College Station, TX, USA) and RevMan 5.3 (The Nordic Cochrane Centre, The Cochrane Collaboration, Copenhagen, Denmark), which measured data using 95% confidence intervals (CIs) and mean differences (MDs). Enumeration was carried out using the hazard ratio (HR) and the odds ratio (OR) and their corresponding 95% CIs. The *I* ^2^ and *p-*values were taken as heterogeneity test indexes. When the *I* ^2^ value was ≤ 50% and the *p-*value was ≥0.1, the heterogeneity between studies was considered to be low, and the fixed-effects model was used. Conversely, when the *I* ^2^ value was > 50% and the *p-*value was < 0.1, some heterogeneity between studies was considered, and the random-effects model was adopted in accordance with the Cochrane Manual. This was followed by an analysis of the source of heterogeneity.

### Quality assessment

The Cochrane risk offset assessment tool was used for the quality assessment.

### Statistical analysis

Our selection of statistical indicators is as follows. The effect quantity that reasonably reflected the overall data was selected in accordance with the data type. The standardized mean difference (SMD) was selected for continuous variable data, and the HR and OR were selected for two-category variable data. For the heterogeneity analysis, after selecting the effect amount, two researchers independently extracted the input data and exchanged and compared the data after completion. After data reconciliation, a meta-analysis was performed using Stata 17.0 and RevMan 5.3, which measured data using 95% CIs and MDs. The *I* ^2^ and *p-*values were taken as heterogeneity test indexes. Subgroup analyses were planned for patients with CKD who had diabetes. For the sensitivity analysis, the included studies were excluded one by one in the Stata 17.0 software to assess the stability of the results. In the published offset evaluation, Begg’s and Peter’s tests could be used for quantitative evaluation. A *p*-value < 0.05 was defined as the publication offset. When there was a publication offset, the cause was found, and the resulting stability was assessed using the shear-complement method.

## Results and analysis

### Literature retrieval results

**Figure 1:**
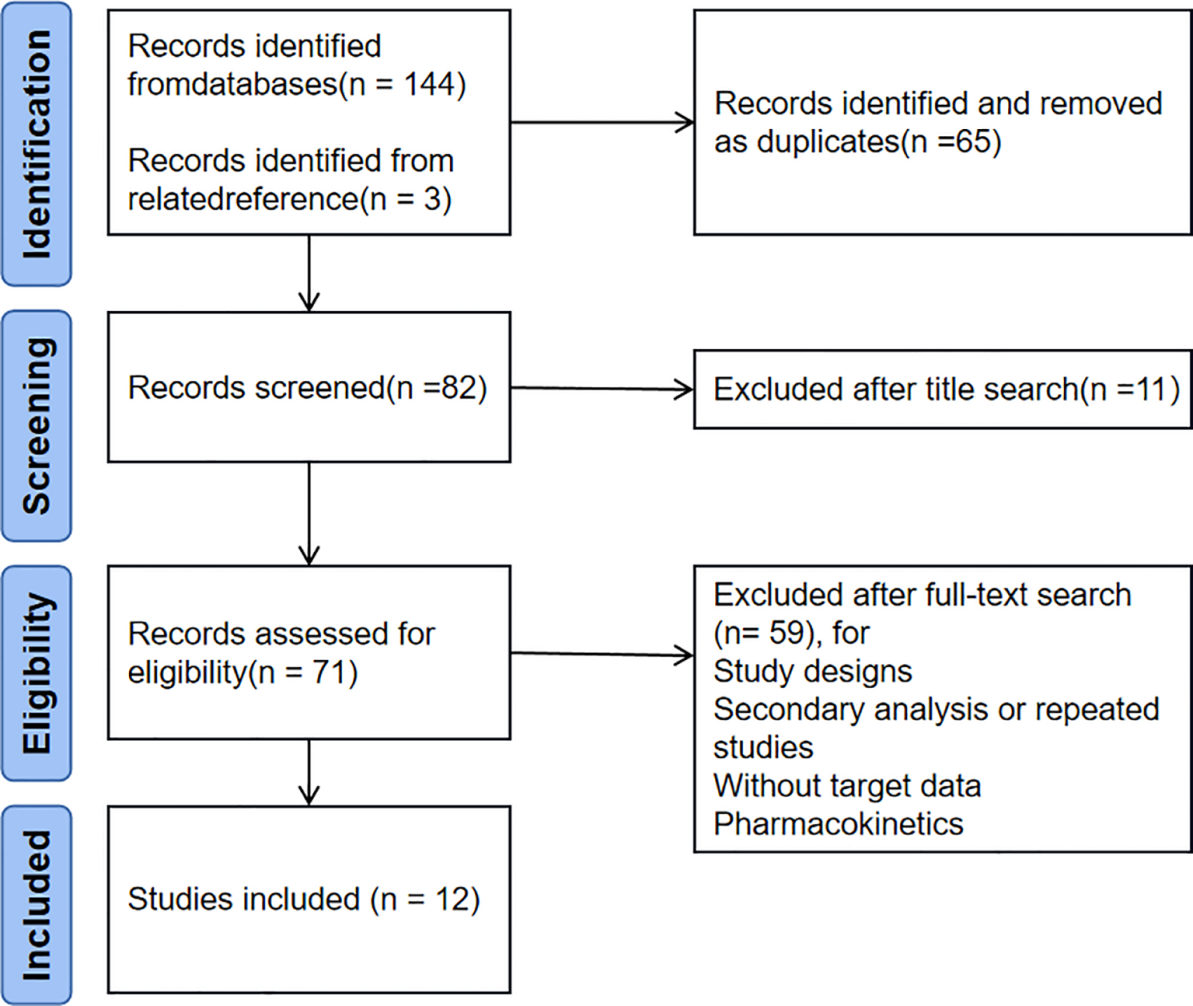
Flow diagram of the selection process.

Each database was searched from the establishment of each database to January 2023 with the search terms DIABETIC NEPHROPATHIES, RANDOMIZED CONTROLLED TRIAL, and SODIUM-GLUCOSE CO-TRANSPORTER 2 INHIBITOR. Keyword retrieval was preferred in all databases, and PubMed retrieval was taken as an example. The search expressions were: (((“Diabetic Nephropathies” [MeSH]) OR “Renal Insufficiency, Chronic” [MeSH])) AND “Randomized Controlled Trial” [Publication Type]) AND (sodium-glucose cotransporter 2 inhibitor OR SGLT 2 inhibitor OR canagliflozin OR dapagliflozin OR empagliflozin OR ertugliflozin OR ipragliflozin OR luseogliflozin OR tofogliflozin OR Sotagliflozin). This improved the recall rate by retrieving relevant references from the American Clinical Research Center and reading the included articles. PubMed retrieved 71 published articles, and Web of Science retrieved 73 articles. Three articles were retrieved from other sources, totaling 147 articles. Use EndNote (Clarvate) or manually delete 65 duplicate articles, and delete 11 unrelated articles based on their titles. Based on the abstracts and full text of other articles, we excluded three study designs, 17 secondary or repeated studies, 37 non targeted study data, and two pharmacokinetic studies. Finally, 12 articles (10, 12–22) were included in the present study. The specific literature screening flow chart is shown in **Figure 1**.

### Basic characteristics and quality evaluation of the inclusion study

**Figure 2:**
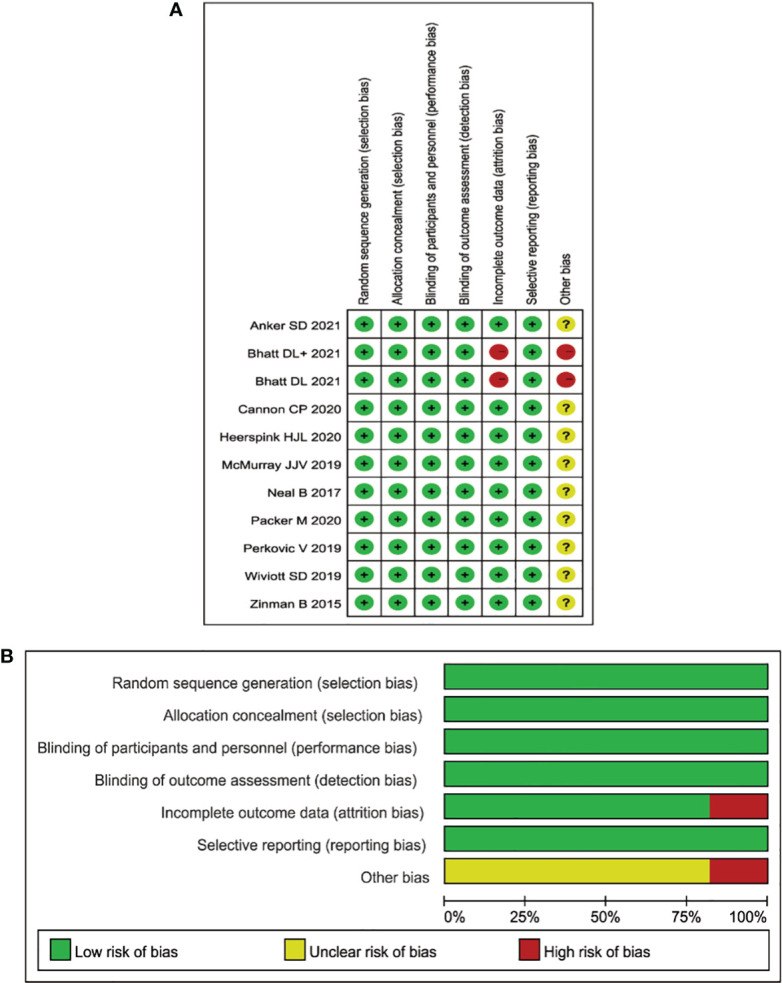
**(A)** Aggregate risk-of-bias graph for each experimental animal study. **(B)** Risk-of-bias summary.

All 12 studies were randomized, controlled, double-blind trials and a Cochrane risk assessment was used to evaluate the risk quality. There were 12 pieces of literature corresponding to 13 RCTs, and each of the studies completely described the method of random distribution and the hidden scheme of distribution. All 12 studies reported the number of patients and their reasons for being lost to follow-up, dropping out, or quitting. The longest follow-up time was 8 years, and the shortest follow-up time was 26 months. The baseline level for the eGFR was reported. See **Figure 2** for the research risk assessment and **Table 1** for the general features.

**Table 1:** Basic characteristics of inclusion study.

Study	Study Design	Sample Size	CKD	Follow-up time	Follow-up Time	eGFR (ml/min/1.73m^2^)	LVEF%
Anker SD 2021 (12)	RCT	5988	YES+NO	Empagliflozin(n=2997) Placebo(n=2991)	Up to 1403 days	eGFR>40	T:54.3±8.8 C:54.3±8.8
Bhatt DL 2021 (17)	RCT	1222	YES	Sotagliflozin(n=608) Placebo(n=614)	Up to 21.6 months	eGFR≥30	T:35 (28–47) C:35 (28–45)
Bhatt DL+ 2021 (18)	RCT	10584	YES	Sotagliflozin(n=5292) Placebo(n=5292)	Up to 30 months	25≤eGFR≤60	NA
Cannon CP 2020 (19)	RCT	8246	YES+NO	Ertugliflozin(n=5499) Placebo(n=2747)	Up to 6 years	eGFR≥30	NA
Heerspink HJL 2020 (20)	RCT	4304	YES	Dapagliflozin(n=2152) Placebo(n=2152)	Up to 38.2 months	25≤eGFR≤75	NA
McMurray JJV 2019 (13)	RCT	4744	YES+NO	Dapagliflozin(n=2373) Placebo(n=2371)	Up to 27.8 months	eGFR≥30	T:31.2±6.7 C:30.9±6.9
Neal B 2017 (21)	RCT	10142	YES+NO	Canagliflozin(n=5795) Placebo(n=4347)	Up to 8 years or about 3 years	eGFR>30	NA
Packer M 2020 (14)	RCT	3730	YES+NO	Empagliflozin(n=1863) Placebo(n=1867)	Up to 1040 days	eGFR≥20	T:27.7±6.0 C:27.2±6.1
Perkovich V 2019 (10)	RCT	4401	YES	Canagliflozin(n=2202) Placebo(n=2199)	Up to 4.6 years	eGFR≥30	NA
The EMPA-KIDNEY Collaborative Group 2022 (16)	RCT	6609	YES	Empagliflozin(n=3304) Placebo(n=3305)	Up to 26 months	20≤eGFR≤90	NA
Wiviott SD 2019 (15)	RCT	17160	YES+NO	Dapagliflozin(n=8582) Placebo(n=8578)	up to 5.2 years	eGFR ≥60	T:85.4±15.8 C:85.1±16.0
Zinman B 2015 (22)	RCT	7028	YES+NO	Empagliflozin(n=4691) Placebo(n=2337)	Up to 4.6 years	eGFR≥30	NA

+The author of this study is the same as the author of the previous study (the same below).

CKD, chronic kidney disease; eGFR, estimated glomerular filtration rate; LVEF, left ventricular ejection fraction; NA, not assessed; RCT, randomized controlled trial.

### Specific data analysis

#### Risks of cardiovascular death or hospitalization for heart failure

**Figure 3:**
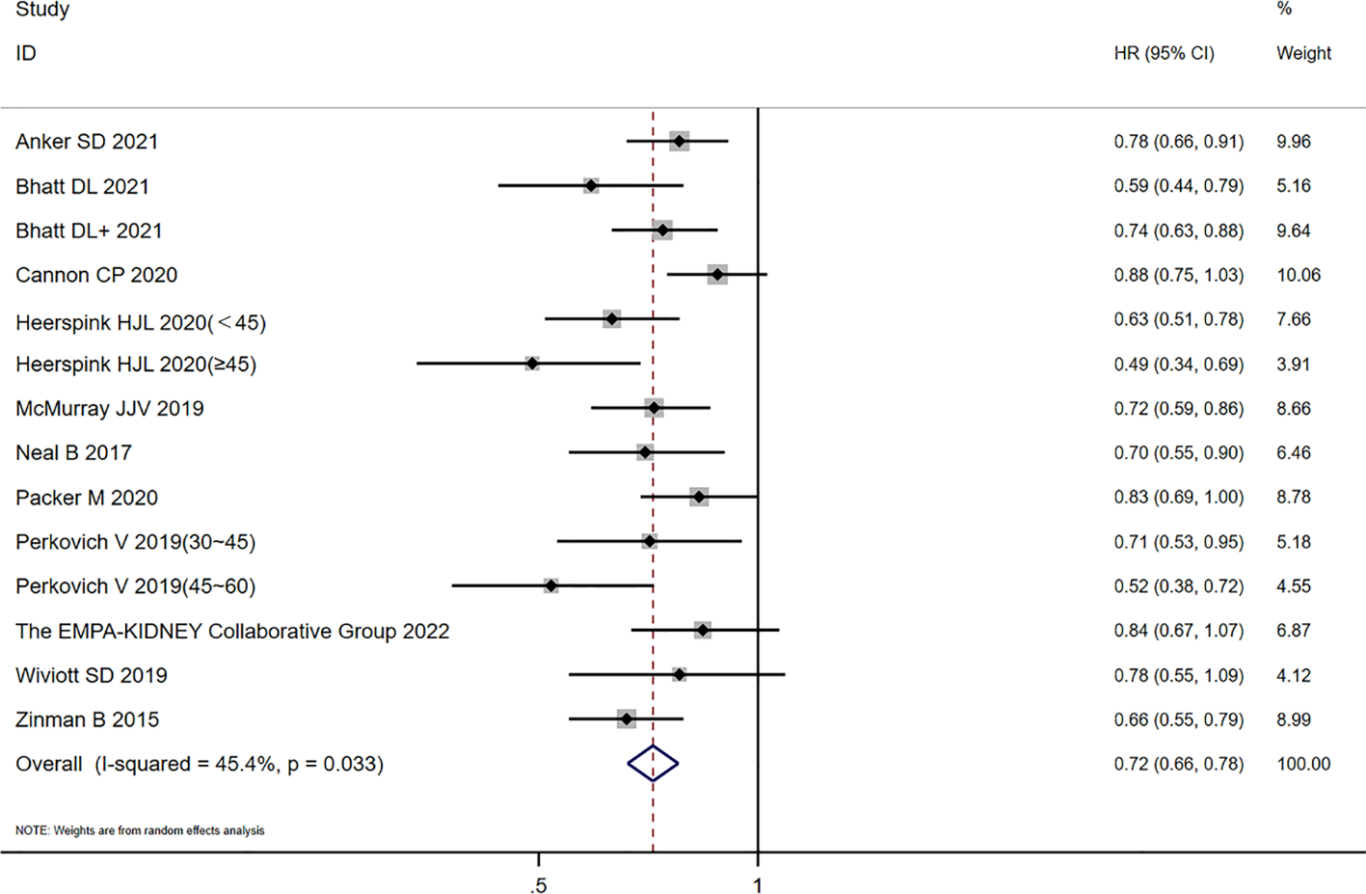
Forest plot of CVD or HHF risk comparisons between SGLT2 inhibitors and placebo in the CKD group. The “+” denotes different studies by the same author and the eGFR subgroups of the same study are shown in parentheses.

There were 12 studies included in this (CKD) analysis: the scope of one study (20) was 25–75 mL/min/1.73m^2^, and seven (10, 15, 17–19, 21, 22) of the 12 studies had diabetes as a complication. The other group (10, 12–15, 17, 21) (with an eGFR ≥ 60 mL/min/1.73m^2^) was included in this study, and all the people in this group had diabetes as a complication. There was heterogeneity among studies for the CKD groups (*I* ^2 ^=^ ^45.4%; *p* = 0.033). The random-effects model was used to analyze the HR values in the 12 studies. Compared with the placebo group, SGLT2 inhibitors reduced the risk of CVD or HHF (HR = 0.72, 95% CI 0.66 to 0.78; *p* = 0.000), and the difference was statistically significant (see **Figure 3**).

Excluding an eGFR of 25–75 mL/min/1.73m^2^, there was no heterogeneity among the studies of the CKD groups (*I* ^2 ^=^ ^33%; *p* = 0.127), and the fixed-effects model (HR = 0.749, 95% CI 0.705 to 0.795; *p* = 0.00) was used. A subgroup analysis of the patients with diabetes showed that there was heterogeneity among the studies (*I* ^2 ^=^ ^46.8%, *p* = 0.068; HR = 0.71, 95% CI 0.63 to 0.79, *p* = 0.000), and there was no obvious additional benefit compared with the mixed population.

In the eGFR ≥ 60 mL/min/1.73m^2^ group, there was no heterogeneity among the studies (*I* ^2 ^=^ ^15.7%; *p* = 0.302). Compared with the placebo group, the SGLT2 inhibitor group reduced the risk of CVD or HHF (HR = 0.82, 95% CI 0.77 to 0.89; *p* = 0.000} with a statistical difference. The diabetes subgroup analysis showed that there was no heterogeneity among the studies (*I* ^2 ^=^ ^0.0%; *p* = 0.697), and the effect value HR was combined with the fixed-effects model, with a statistical difference (HR = 0.88, 95% CI 0.80 to 0.97; *p* = 0.008]. In summary, in the diabetes subgroup analysis, the SGLT2 inhibitor group reduced the risk of CVD and HHF.

#### Risks of cardiovascular death

**Figure 4:**
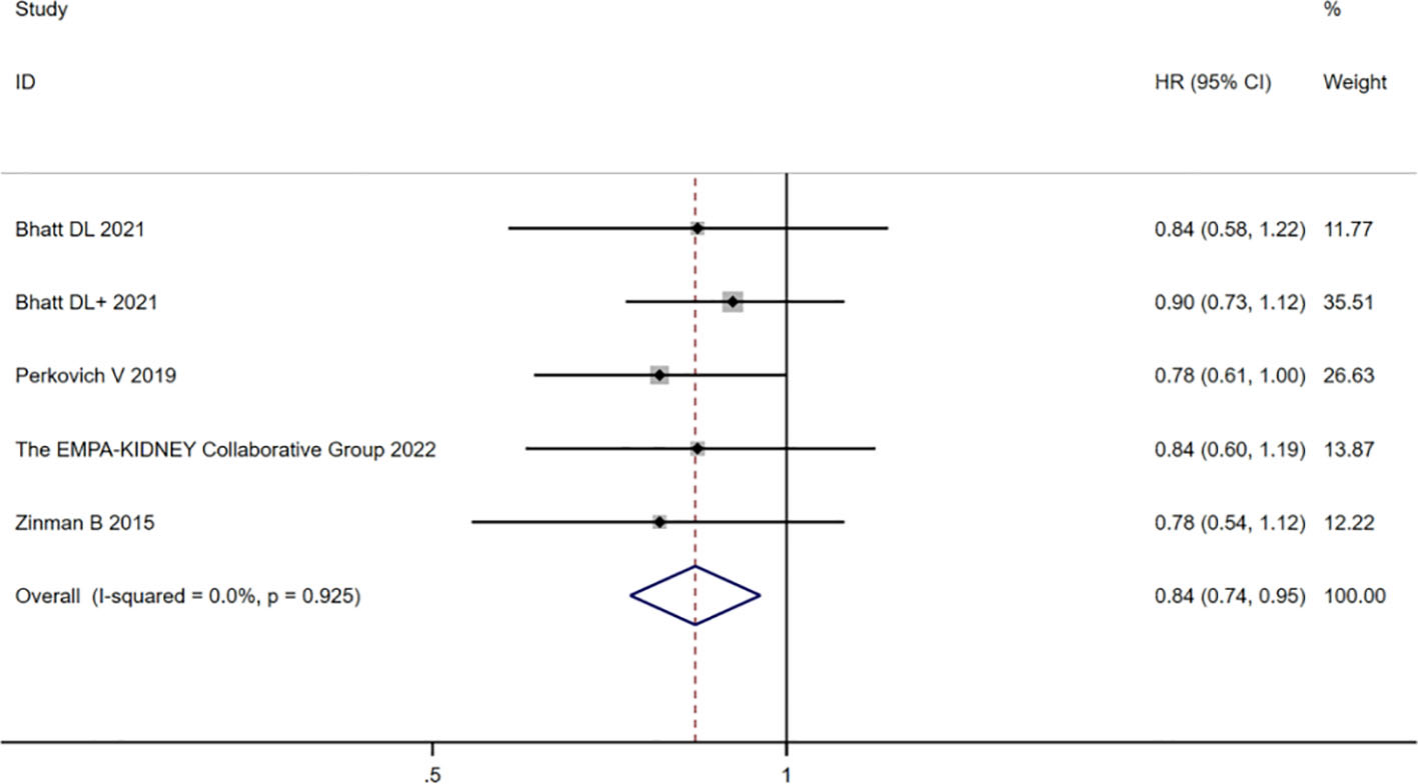
Forest plot of the risk of cardiovascular death in the CKD group, SGLT2 inhibitors, and placebo. The “+” denotes different studies by the same author and the eGFR subgroups of the same study are shown in parentheses.

Among the 12 studies included, five (10, 16–18, 22) described CVD in the CKD population. The heterogeneity of CVD risk among the 12 studies was very low (*I* ^2 ^=^ ^13.3%; *p* = 0.314). The combined analysis of HR values by using the fixed-effects model showed that the risk of CVD in the SGLT2 inhibitor group had a value of HR equal to 0.86 (95% CI 0.81 to 0.92; *p*=0.000) compared with the placebo group, which was statistically significant. The heterogeneity of the CKD group was very low (*I* ^2 ^=^ ^0%; *p* = 0.925), and the analyzed HR was 0.84 (95% CI 0.74 to 0.95; *p* = 0.006], which was still statistically significant, as shown in **Figure 4**. In summary, the SGLT2 inhibitor group reduced the risk of CVD in patients with or without CKD.

#### Risks of hospitalization for heart failure

Ten (10, 12–15, 17–19, 21, 22) of the 12 studies included HHF data, and three (10, 17, 18) of the studies described HHF in the CKD population. The heterogeneity among the studies was very low (*I* ^2 ^=^ ^0%; *p* = 0.993). If the fixed-effects model was used for the combination analysis of HR values, the risk of HHF in the SGLT2 inhibitor group, compared with that in the placebo group, had a value of HR equal to 0.68 (95% CI 0.64 to 0.73; *p* = 0.000), which was statistically significant. In the hospitalization risk study for heart failure in patients with CKD, the heterogeneity was very low (*I* ^2 ^=^ ^0%; *p* = 0.856), and the analyzed HR was 0.65 (95% CI 0.56 to 0.74; *p* = 0.000), which was still statistically significant. In summary, SGLT2 inhibitors reduced the risk of HHF in patients with or without CKD.

#### Risk of all-cause death

Five (10, 12, 16, 17, 20) of the 12 included studies described all-cause death in the CKD population. There was significant heterogeneity among the studies (*I* ^2 ^=^ ^41.6%; *p* = 0.064). When the HR values were combined and analyzed using the random-effects model, compared with the placebo group, the risk of all-cause death with SGLT2 inhibitors had a value of HR equal to 0.87 (95% CI 0.82 to 0.93; *p* = 0.0000), which had statistical significance. In the all-cause death risk study for CKD patients, the heterogeneity was very low (*I* ^2 ^=^ ^28.2%; *p* = 0.234), and the analyzed HR was 0.86 (95% CI 0.78 to 0.95; *p* = 0.003), which was statistically significant. In summary, SGLT2 inhibitors reduced the risk of all**-**cause death in patients with or without CKD.

### Serious adverse events

Ten (10, 12–15, 17–20, 22) of the 12 studies contained data for SAEs, and four (10, 17, 18, 20) of them described SAEs in the CKD population. A total of 67,407 patients were included, including 36,259 in the SGLT2 inhibitor group and 31,148 in the placebo group. The heterogeneity among studies was extremely low (*I* ^2 ^=^ ^0%, *p* = 0.98). The fixed-effects model was applied to merge the effect values’ OR using the Mantel–Haenszel (M–H) method. Compared with the placebo group, the incidence of SAEs in the SGLT2 inhibitor group was relatively low (OR = 0.88, 95% CI 0.85 to 0.91; *p* < 0.0001), which was statistically different, in which the risk of SAEs in CKD patients was very low (*I* ^2^ = 0%; *p* = 0.58).

### Reproductive tract infections

Nine (10, 12, 14–20) of the 12 studies included data on RTIs, and five (10, 16–18, 20) of the studies described RTIs in the CKD population. A total of 62,244 patients were included, including 32,499 cases in the SGLT2 inhibitor group and 29,745 cases in the placebo group. There was low heterogeneity among studies (*I* ^2 ^=^ ^31.3%; *p* = 0.22). The fixed-effects model was used, and the M–H method was used to combine the effect values’ OR. The SGLT2 inhibitor group had a higher incidence of RTIs than the placebo group (OR = 3.56, 95% CI 2.96 to 4.27; *p* < 0.0001), which was statistically different. In the study on the risk of RTIs in patients with CKD, the heterogeneity was very low (I^2 ^=^ ^0%; *p* = 0.80), and the analyzed OR was 3.06 (95% CI 2.29 to 4.10; *p* < 0.0001), which was still statistically significant. That is, the SGLT2 inhibitors increased the risk of RTIs in patients with or without CKD.

### Urinary tract infections

Eleven (10, 12–20, 22) of the 12 studies contain data on UTIs, and five (10, 16–18, 20) of them describe UTIs in the CKD population. A total of 74,016 patients were included, including 39,563 cases in the SGLT2 inhibitor group and 34,453 cases in the placebo group. The heterogeneity among studies was extremely low (*I* ^2 ^=^ ^20.7%; *p* = 0.26). The fixed-effects model was applied, and the M–H method was selected to merge the effect values’ OR. Compared with the placebo group, the incidence of UTIs in the SGLT2 inhibitor group had an OR equal to 1.10 (95% CI 1.03 to 1.18; *p* = 0.004), and there was no statistical difference. Among them, the risk of UTIs in CKD patients was very low (*I* ^2 ^=^ ^0%; *p* = 0.87), and the OR was 1.06 (95% CI 0.96 to 1.17; *p* = 0.22), which was not statistically different. In summary, the SGLT2 inhibitors have no effect on the risk of UTIs in patients with or without CKD.

### Publication bias analysis

**Figure 5:**
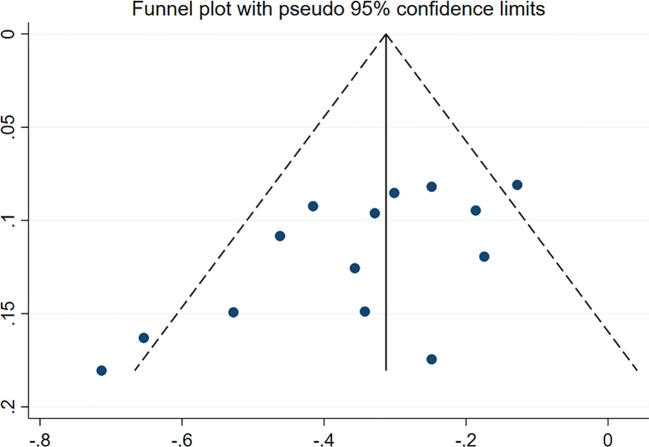
CVD/HHF funnel diagram of the CKD group.

More than 10 studies were included in the outcome index. In addition, the funnel chart was drawn using Stata 17.0. As shown in **Figure 5**, a CVD/HHF risk funnel diagram of patients in the CKD group was drawn. The other groups contained fewer than 10 articles, and the publication deviation was evaluated by Begg’s test or Peter’s test with Stata 17.0. Apart from the Egger test of the CVD/HHF results in the CKD group (*p* = 0.013 < 0.05), which indicated that there was significant bias, no significant publication bias was observed in any results (*p* > 0.05). Therefore, the results of CVD or HHF in the CKD group were subjected to shear supplementary correction to obtain no additional studies, and the results still had a value of HR equal to 0.72 (95% CI 0.66 to 0.78; *p* = 0.000). However, after deleting the studies with an eGFR range of ≥ 25 mL/min/1.73m^2^ to ≤ 75 mL/min/1.73m^2^, the Egger test has no significant publication bias.

### Sensitivity analysis

STATA 17.0 was used to analyze the sensitivity of the outcome indicators. After all the studies of the outcome indicators were eliminated one by one, the combined effect amount did not change significantly, suggesting that the cardiovascular outcome indicators and adverse reaction indicators were stable.

## Discussion

CKD is a chronic progressive disease that is related to many CVDs other than heart disease, including stroke, peripheral arterial disease, aortic aneurysm, and venous thrombosis (23–26). In the cohort study, people with low eGFR or CKD but no elevated urine albumin-to-creatinine ratio had a higher independent relative death risk. Similar results were observed for cardiovascular-specific mortality (27). CKD is usually defined as low eGFR or increased proteinuria. CKD affects 10% to 16% of the adult population in Asia, Australia, Europe, and the USA (28). It increases the risk of various adverse consequences. The 2020 Global Burden of Disease study identified CKD as one of the top 10 causes of poor prognosis in the world (29). Among the main adverse consequences related to CKD, CVD is one of the most important diseases because it is one of the main causes of death in this clinical population.

A lot of evidence shows that CKD is related to many CVD results other than heart disease, including coronary heart disease (30), stroke (31), heart failure (32), peripheral arterial disease (24), abdominal aortic aneurysm (26), and venous thrombosis (33). In several studies, CKD has been strongly correlated with sporadic or generalized atrial fibrillation (34, 35). It is reported that CKD is related to sudden cardiac death (36, 37). Patients with CKD often have atherosclerotic dyslipidemia, which is characterized by an increase in triglyceride and low-density lipoprotein cholesterol levels and a decrease in high-density lipoprotein cholesterol levels (38).

The results of this study show that SGLT2 inhibitors can reduce the risk of CVD in CKD patients, especially in reducing the risk of HHF. At the same time, SGLT2 inhibitors also reduced the risk of CVD or HHF in the non-CKD group. The benefits in a mixed population and a diabetic population were also compared, and the results showed that there was no obvious difference between the two groups. At present, the molecular mechanism of improving cardiovascular and renal function is unclear. Studies have shown that SGLT2 inhibitors can improve urinary protein and delay the progress of CKD mainly by inhibiting the SGLT2 receptor in renal tubules, inhibiting the reabsorption of sodium ions and glucose, contracting into the glomerular arteriole through the tubuloglomerular balance, and reducing the pressure on the glomerulus (39).

SGLT2 inhibitors have been shown to provide remarkable benefits in the clinical study of CKD patients. Many studies have shown that SGLT2 inhibitors can reduce the risk of serious cardiac and renal outcomes in patients (10, 11, 16, 18), improve the patient’s cardiac and renal outcomes, and reduce the number of hospitalizations (9, 10), thereby reducing the medical expenses in this respect. SGLT2 inhibitors can also reduce the incidence of hyperkalemia in CKD patients (40).

SGLT2 inhibitors may play their role by controlling the energy metabolism pathway *in vivo* through urine glucose excretion and by promoting cell apoptosis, resisting autophagy, upregulating cell repair mechanisms, inducing cell anti-stress abilities, reducing vascular inflammation and arterial stiffness, inhibiting the reabsorption of sodium, and reducing the load of body fluids. At the same time, the decrease in urinary protein, systolic blood pressure, and diastolic blood pressure levels is also related to the decrease in cardiovascular risk.

CKD often needs comprehensive treatment, often combined with angiotensin-converting enzyme inhibitors, angiotensin receptor blockers, beta-blockers, and other drugs. Because SGLT2 inhibitors contract into the bulbar arteriole through tubuloglomerular feedback, the combination of angiotensin-converting enzyme inhibitors or angiotensin receptor blockers may increase the risk of acute renal injury. A retrospective analysis of a clinical study found that SGLT2 inhibitors combined with angiotensin-converting enzyme inhibitors or angiotensin receptor blockers still had the function of protecting renal function, compared with the group without SGLT2 inhibitors, and did not increase the incidence of acute renal adverse events in CKD patients (41). SGLT2 inhibitors combined with angiotensin-converting enzyme inhibitors can up-regulate the renin–angiotensin system in CKD patients (7, 8). Because SGLT2 inhibitors can lower blood sugar levels, a meta-analysis of angiotensin-converting enzyme inhibitors or angiotensin receptor blockers combined with SGLT2 inhibitors in the treatment of diabetes found that combined therapy increased the risk of hypoglycemia (42). Therefore, attention should be paid to the possibility of hypoglycemia during treatment.

In this study, SGLT2 inhibitors did not increase the incidence of SAEs or UTIs, but they did increase the incidence of genital infections. Multiple studies share similar perspectives with this study. This study has shown that SGLT2 inhibitors exposure is not associated with an increased risk of UTIs (43). No difference was found in UTI incidence when comparing SGLT2 inhibitors with placebo in patients (44). Another study has shown that SGLT2is does not have an increased risk of genitourinary infections compared with metformin (45). A retrospective study found that SGLT2 inhibitors medications were not commonly initiated in the 6 months prior to the occurrence of a UTI (46). The results of another study are consistent with our conclusion about genital infections with SGLT2 inhibitors, and most of the reported infections responded to standard treatment (47). This study also found that, apart from SGLT2 inhibitors, factors including personal hygiene, menopause, and circumcision might have a possible role in reported events of genital infection among T2DM patients on SGLT2 inhibitors therapy. Similar to the results of another safety analysis study of SGLT2 inhibitors, the results of this study showed that the drug has a higher risk of non-spinal fracture, lower limb amputation, genital infection, diabetic ketoacidosis, hypovolemia, hypoglycemia, and severe UTIs, with similar risks; however, SGLT2 inhibitors have a lower risk of acute kidney injury (48). Furthermore, another study found that the use of SGLT2 inhibitors would lead to a slight increase in the rate of fungal UTIs (49). It is inconsistent with the conclusion of this study that SGLT2 inhibitors will not increase the probability of UTIs.

Studies have shown that the conclusions are different with the use of different types of SGLT2 inhibitors. Compared with placebo and other active treatments, canagliflozin, dapagliflozin, and empagliflozin are associated with significantly increased risks of genital infections. Only dapagliflozin has a dose–response relationship with UTIs and genital infections (50). This may be related to the early decline of the eGFR when SGLT2 inhibitor treatment started; however, the follow-up data showed that the eGFR returned to near the baseline, and the decline of the eGFR was no different from that of the placebo (51), or the slowdown of the decline of the eGFR compared with the control group (20, 52). Some studies have also found that SGLT2 inhibitors have statistical significance in alleviating the decline slope of the eGFR (53, 54). This is because the amount of liquid and NaCl delivered to the distal renal tubule increases, and the glomerular filtration rate is reduced by tubuloglomerular feedback (55). When combined with loop diuretics, SGLT2 inhibitors resulted in a significant increase in 24-hour urine volume, while urinary sodium levels did not increase. The possible sodium benefits of SGLT2 inhibitors may be short-lived and only appear in the early stages (56). It is consistent with the changing trend of the eGFR.

## Conclusion

SGLT2 inhibitors can protect the cardiovascular system of CKD patients and reduce the risk of CVD or HHF. Compared with placebo, the risk of CVD, HHF, and all-cause death are all reduced, and the use of SGLT2 inhibitors does not increase the incidence of SAEs or UTIs but may increase the incidence of genital infections.

## Data availability statement

The original contributions presented in the study are included in the article/Supplementary Material. Further inquiries can be directed to the corresponding author.

## Author contributions

TZ contributed to the conception and design of the study. XC and JW were responsible for the collection of data, performing the statistical analysis, and manuscript preparation. YL, KY, and YX were responsible for checking the data. XC was responsible for drafting the manuscript. All authors contributed to the article and approved the submitted version.
